# Lack of Evidence That Selenium-Yeast Improves Chicken Health and Modulates the Caecal Microbiota in the Context of Colonization by *Campylobacter jejuni*

**DOI:** 10.3389/fmicb.2017.00451

**Published:** 2017-03-17

**Authors:** Alexandre Thibodeau, Ann Letellier, Étienne Yergeau, Guillaume Larrivière-Gauthier, Philippe Fravalo

**Affiliations:** ^1^Faculty of Veterinary Medicine, University of Montreal – NSERC Industrial Research Chair in Meat Safety, Saint-HyacintheQC, Canada; ^2^INRS–Institut Armand-Frappier Research Centre, Université du Québec, LavalQC, Canada

**Keywords:** chicken, selenium, *Campylobacter jejuni*, microbiota, health

## Abstract

Faced with ever-increasing demand, the industrial production of food animals is under pressure to increase its production. In order to keep productivity, quality, and safety standards up while reducing the use of antibiotics, farmers are seeking new feed additives. In chicken production, one of these additives is selenium. This element is expected to confer some advantages in terms of animal health and productivity, but its impact on chicken intestinal microbiota as well as on the carriage of foodborne pathogens is unknown. In this study, chickens raised in a level 2 animal facility were fed or not 0.3 ppm of in-feed selenium-yeast until 35 days of age and were inoculated or not with the foodborne pathogen *Campylobacter jejuni* at the age of 14 days. At the end of the study, body weight, seric IgY, intestinal IgA, seric gluthatione peroxydase activity, the caecal microbiota (analyzed by MiSeq 16S rRNA gene sequencing), and *C. jejuni* caecal levels were analyzed. The experiment was completely replicated twice, with two independent batches of chickens. This study revealed that, for healthy chickens raised in very good hygienic conditions, selenium-yeast does not influence the bird’s body weight and lowers their seric gluthatione peroxidase activity as well as their intestinal IgA concentrations. Furthermore, selenium-yeast did not modify the caecal microbiota or the colonization of *C. jejuni*. The results also showed that *C. jejuni* colonization does not impact any of the measured chicken health parameters and only slightly impacts the caecal microbiota. This study also clearly illustrated the need for true biological replication (independent animal trials) when assessing the microbiota shifts associated with treatments as the chickens microbiotas clearly clustered according to study replicate.

## Introduction

Worldwide, the demand for food is increasing at an exponential rate (National Academy of Science, 2015), putting an ever-increasing pressure on meat production. In Canada, compared to 1991, beef and pork consumption *per capita* is declining while chicken consumption is increasing (Agriculture and Agrifood Canada [AAFC], 2016). Chicken meat production is following the same trend; in Canada, 610 million live birds were produced in 2005 compared to 661 million in 2015 (Agriculture and Agrifood Canada [AAFC], 2016). This increased production is challenging the food production system. In response to these challenges, the “One Health” approach – which recognizes and puts emphasis on the direct link between production animal health and public health – is gaining momentum in animal husbandry. For example, in Canada, the on-farm use of antibiotics is becoming more controlled in an effort to promote the judicious use of antibiotics (Gazette du Canada, 2016) and surveillance of foodborne pathogens is being tightened (Canadian Food Inspection Agency [CFIA], 2016a). Therefore, to continue the production of high-quality chicken proteins while ensuring the lowest possible contamination of products by foodborne pathogens, producers must adapt their rearing practices. One avenue is to modify animal feed composition by the inclusion of selected feed additives aimed at increasing the health of chicken flocks.

One of these feed additives is selenium. Selenium can be added to chicken feed in the organic or inorganic form (Zoidis et al., 2014). It has been observed that selenium in-feed supplementation increases carcass weight, oxidative stress response, immune response and it appears to be most effective in limiting morbidity when the broilers are exposed to disease or environmental stress (Zhou and Wang, 2011; Chen et al., 2014; Markovic et al., 2014; Boostani et al., 2015; Xu et al., 2015; Sun et al., 2016). One interesting form of selenium that is commercially available is yeast grown in a media enriched with selenium. This selenium-enriched yeast provides a source of organic selenium to chickens. Furthermore, yeast has been observed to be a good feed additive for chickens as they may act as a probiotic (Caly et al., 2015; Mountzouris et al., 2015; Nawaz et al., 2016), making selenium-yeast an interesting product. The impact of selenium on healthy chickens is not fully characterized while its potential impact on chicken intestinal health, in particularly the intestinal microbiota, remains poorly characterized.

The chicken microbiota is defined as the microorganism community that inhabits the chicken (Oakley et al., 2014). The caecal microbiota is becoming increasingly characterized and was observed to be modulated by a plethora of factors, ranging from feed composition to disease (Thibodeau et al., 2015b; Ajuwon, 2016; Antonissen et al., 2016). An optimal microbiota, conferring optimal health and growth, has yet to be defined in chickens, illustrating the need for deeper research in this field. One particularly interesting member of the chicken microbiota is one that often colonizes the chicken caecum and is a zoonotic foodborne pathogen for humans: *Campylobacter jejuni* (Chen and Jiang, 2014).

*Campylobacter jejuni* is the most common bacterial foodborne pathogen worldwide (Kirk et al., 2015). It colonizes the chicken caecum at very high loads and therefore easily contaminates chicken food products during processing (Meunier et al., 2016). Reducing chicken *C. jejuni* caecal carriage would lessen the health burden associated with this particular pathogen (Meunier et al., 2016). *C. jejuni* colonization of chickens was observed to be strain dependent and to be mediated by colonization genes that have yet to be completely identified (Thibodeau et al., 2015a). *C. jejuni* mainly uses amino acids and organic acids for its energy production (Hofreuter, 2014). It has been shown that *C. jejuni* colonization can be reduced by competitive exclusion caused by modifications to the chicken microbiota (Laisney et al., 2004). Food additives that could modify the chicken microbiota, such as selenium-yeast, are of particular interest in that context.

Consequently, the aim of this study was to assess the effect of selenium-yeast in-feed supplementation on broiler chicken health parameters and on the caecal microbiota, with a specific focus on *C. jejuni* colonization.

## Materials and Methods

### *In vivo* Chicken Experimentations

All animal experimentations were approved by the ethics committee of the Faculty of Veterinary Medicine of the University of Montreal, certificate number 14-Rech-1730. Newly hatched broiler chickens (Ross 308) were purchased at a local hatchery and transported to the avian research center (level 2 confinement facility) of the veterinary medicine faculty. All chickens were vaccinated at the hatchery against Marek’s disease and infectious bronchiolitis. The exact number of analyzed chickens per group per experiment is presented in each table (**Tables 1**–**7**). Chickens were placed in two different rooms: the chickens housed in the first room were inoculated with *C. jejuni* while the chickens housed in the second room were not to be inoculated with *C. jejuni*. The chickens housed in each room were further separated into two groups: one group received an in-feed supplementation of a selenium-yeast commercial preparation at 0.3 ppm and the other did not. This selenium-yeast concentration is the maximum supplementation allowed by the Canadian Food Inspection Agency (Canadian Food Inspection Agency [CFIA], 2016b). All chickens were fed a standard mash diet and had *ad libitum* access to water and feed.

**Table 1:** Observed mean body weight (g) for chickens according to the use of selenium-yeast or the inoculation of *C. jejuni*.

Condition	In-feed selenium-yeast		*C. jejuni* inoculation	
		
	No additive	Selenium-yeast	*C. jejuni* neg	*C. jejuni* pos
Replicate 1	2190 (160.7)	2249 (111.9)	2202 (128.1)	2235 (151.1)
Replicate 2	2190 (221.7)	2248 (214)	2254 (202.5)	2186 (230)
Replicate 1+2	2190 (190.3)	2248 (168.6)	2248 (169.2)	2211 (192.8)


*Standard deviation reported in parenthesis. Replicate 1+2: combined analysis of biological replicates. Chickens analyzed per group for Replicate 1: No additive/*C. jejuni* neg = 9, No additive/*C. jejuni* pos = 11, Selenium-yeast/*C. jejuni* neg = 10, Selenium-yeast/*C. jejuni* pos = 10. Chickens per group for Replicate 2: No additive/*C. jejuni neg* = 10, No additive/*C. jejuni* pos = 9, Selenium-yeast/*C. jejuni* neg = 9, Selenium-yeast/*C. jejuni* pos = 11.*

**Table 2:** Observed seric GPX activity (U/ml) for chickens according to the use of selenium-yeast or the inoculation of *C. jejuni*.

Condition	In-feed selenium-yeast		*C. jejuni* inoculation	
		
	No additive	Selenium-yeast	*C. jejuni* neg	*C. jejuni* pos
Replicate 1	1532 (996) ***^∗^a***	680 (523) ***^∗^b***	1275 (1194)	1073 (354)
Replicate 2	1264 (673) ***^∗^a***	825 (845) ***^∗^b***	1131 (750)	958 (830)
Replicate 1+2	1394 (843) ***^∗^a***	755 (701) ***^∗^b***	1202 (993)	949 (662)


**Table 3:** Seric IgY concentrations (μg/ml) for chickens according to the use of selenium-yeast or the inoculation of *C. jejuni*.

Condition	In-feed selenium-yeast		*C. jejuni* inoculation	
		
	No additive	Selenium-yeast	*C. jejuni* neg	*C. jejuni* pos
Replicate 1	1.68 (0.80)	2.24 (0.92)	2.01 (1.01)	1.91 (0.79)
Replicate 2	1.59 (0.66)	1.59 (1.12)	1.28 (0.74)	1.90 (0.96)
Replicate 1+2	1.64 (0.72)	1.92 (1.06)	1.64 (0.95)	1.91 (0.87)


*Standard deviation reported in parenthesis. Replicate 1+2: combined analysis of biological replicates. Chickens analyzed per group for Replicate 1: No additive/*C. jejuni* neg = 8, No additive/*C. jejuni* pos = 8, Selenium-yeast/*C. jejuni* neg = 8, Selenium-yeast/*C. jejuni* pos = 8. Chickens per group for Replicate 2: No additive/*C. jejuni neg* = 8, No additive/*C. jejuni* pos = 8, Selenium-yeast/*C. jejuni* neg = 8, Selenium-yeast/*C. jejuni* pos = 8.*

**Table 4:** Observed mean intestinal IgA concentrations (μg/ml) for chickens according to the use of selenium-yeast or the inoculation of *C. jejuni*.

Condition	In-feed selenium-yeast		*C. jejuni* inoculation	
		
	No additive	Selenium-yeast	*C. jejuni* neg	*C. jejuni* pos
Replicate 1	3.83 (2.39)	3.19 (1.36)	2.96 (1.39)	4.06 (2.28)
Replicate 2	7.10 (5.83) ***^∗^a***	3.49 (2.27) ***^∗^b***	3.55 (3.0) ***^∗^c***	7.03 (5.54) ***^∗^d***
Replicate 1+2	5.47 (4.69) ***^∗^a***	3.34 (1.85) ***^∗^b***	3.26 (2.32) ***^∗^c***	5.54 (4.43) ***^∗^d***


**Table 5:** Comparison of identified differences in diversity metrics regarding the use of selenium-yeast, *C. jejuni* inoculation or the study’s replicate.

Replicate	Replicate 1				Replicate 2			

Condition	Selenium-yeast		*C. jejuni*		Selenium-yeast		*C. jejuni*	
				
	Neg	Pos	Neg	Pos	Neg	Pos	Neg	Pos
Coverage	0.98 (0.01)	0.98 (0.01)	0.98 (0.01)	0.98 (0.01)	0.986 (0.005) **^∗^a**	0.982 (0.006) **^∗^b**	0.97 (0.01)	0.97 (0.01)
Sobs	394 (100)	498 (149)	454 (153)	415 (106)	604 (201) **^∗^a**	755 (231) **^∗^b**	691 (257)	684 (208)
Simpson inverse	16.4 (2.6)	15.4 (3.5)	15.6 (3.3)	16.3 (2.8)	14.4 (4.5)	16.2 (6.4)	15.2 (4.8)	15.6 (6.4)
Shannon	3.5 (0.1)	3.5 (0.2)	3.5 (0.2)	3.5 (0.1)	3.5 (0.2)	3.7 (0.3)	3.6 (0.3)	3.6 (0.2)
Shannon even	0.59 (0.03)	0.58 (0.03)	0.58 (0.06)	0.58 (0.06)	0.60 (0.04)	0.60 (0.04)	0.56 (0.04)	0.56 (0.03)


*Condition’s effect on the metric for selenium and *C. jejuni* inoculation; On the same row, ^∗^**a** different from ^∗^**b**; Neg: negative control, Pos: positive; standard deviation in parenthesis. Chickens analyzed per group for Replicate 1: No additive/*C. jejuni* neg = 8, No additive/*C. jejuni* pos = 8, Selenium-yeast/*C. jejuni* neg = 8, Selenium-yeast/*C. jejuni* pos = 7. Chickens per group for Replicate 2: No additive/*C. jejuni neg* = 5, No additive/*C. jejuni* pos = 8, Selenium-yeast/*C. jejuni* neg = 8, Selenium-yeast/*C. jejuni* pos = 8.*

**Table 6:** Common OTUs associated with *C. jejuni* inoculation in both replicates.

*C. jejuni*	OTU classification	LDA score Replicate 1	LDA score Replicate 2
Neg	*B(100);F*(100)*;C*(100)*;Cles*(100)*;Lachno*(100)*;Eisenbergiella*(92)	4.2	4.2
	*B*(100)*;F*(100)*;C*(100)*;Cles*(100)*;Lachno*(100)*;Tyzzerella_3*(100)	2.9	2.6
	*B*(100)*;F*(100)*;C*(100)*;Cles*(100)*;Lachno*(99)*;[Eubacterium]_hallii_group*(84)	3.7	3.8
	*B*(100)*;F*(100)*;C*(100)*;Cles*(100)*;Ruminoc*(100)	3.3	3.0
	*B*(100)*;F*(100)*;C*(100)*;Cles*(100)*;Ruminoc*(100)*;Ruminoc_UCG-014*(100)	3.8	3.7
	*B*(100)*;F*(100)*;C*(100)*;Cles*(100)*;Ruminoc*(99)	4.1	3.8
	*B*(100)*;F*(100)*;C*(100)*;Cles*(100)*;Ruminoc(99)Anaerotruncus*(98)	2.7	2.6
Pos	*B*(100)*;F*(100)*;C*(100)*;Cles*(100)*;Cles_vadinBB60_group*(100)	2.9	3.0
	*B*(100)*;F*(100)*;C*(100)*;Cles*(100)*;Lachno*(100)	4.1	4.4
	*B*(100)*;F*(100)*;C*(100)*;Cles*(100)*;Lachno*(100)	3.0	3.4
	*B*(100)*;F*(100)*;C*(100)*;Cles*(100)*;Lachno*(100)	3.1	2.8
	*B* (100)*;F*(100)*;C*(100)*;Cles*(100)*;Lachno*(100)	3.1	2.5
	*B* (100)*;F*(100)*;C*(100)*;Cles*(100)*;Lachno*(100)	2.3	2.8
	*B* (100)*;F*(100)*;C*(100)*;Cles*(100)*;Ruminoc*(100)*;Anaerotruncus*(98)	4.0	3.5
	*B* (100)*;F*(100)*;C*(100)*;Cles*(100)*;Ruminoc*(100)*;Oscillibacter*(99)	3.4	4.0
	*B*(100)*;F* (100)*;C*(100)*;Cles*(100)*;Ruminoc*(100)*;Ruminoc_UCG-14*(100)	2.0	2.1
	*B*(100)*;ProteoB*(100)*;EpsilonproteoB*(100)*;Campyr*(100)*;Campylo*(100)*;Campylobacter*(100)	4.5	3.5


*The last returned identification for common OTUs is displayed; (): percentage of classification; OTU classification by increased alphabetical order; *B = Bacteria; F = Firmicutes; C = Clostridia; Cles = Clostridiales; Lachno = Lachnospiraceae; Ruminoc = Ruminococcaceae, Campyr = Campylobacterales; Campylo = Campylobacteraceae*; LDA: Linear Discriminant Analysis to estimate the effect size of each differentially abundant feature. Chickens analyzed per group for Replicate 1: No Additive/*C. jejuni* neg = 8, No Additive/*C. jejuni* pos = 8, Selenium-yeast/*C. jejuni* neg = 8, Selenium-yeast/*C. jejuni* pos = 7. Chickens analyzed per group for Replicate 2: No Additive/*C. jejuni* neg = 5, No Additive/*C. jejuni* pos = 8, Selenium-yeast/*C. jejuni* neg = 8, Selenium-yeast/*C. jejuni* pos = 8.*

**Table 7:** Observed *C. jejuni* caecal colonization levels (log 10) for chickens according to the use of selenium-yeast.

Condition	In-feed selenium-yeast	
	
	No additive	Selenium-yeast
Replicate 1	7.1 (0.63) ***^∗^a***	7.8 (0.51) ***^∗^b***
Replicate 2	6.0 (0.71) ***^∗^a***	5.2 (0.60) ***^∗^b***
Replicate 1+2	6.57 (0.84)	6.43 (1.41)


At day 12, fresh caecal droppings were collected from each group to confirm the absence of *C. jejuni* colonization. To differentiate the effect of selenium-yeast from the eventual effect of *C. jejuni* carriage on the chicken health parameters evaluated, at 14 days of age, one room was inoculated with an oral suspension of two deeply characterized *C. jejuni* strains (A2008a and G2008b) (Thibodeau et al., 2013, 2015a,b) while the other was not. The oral suspension was obtained from an overnight blood agar culture of each strain that was suspended in PBS phosphate buffered saline (PBS) to an optic density of 1.0 (at 630 nm) and further diluted to obtain a final concentration of 10^4^ CFU per strain per inoculation.

At 35 days of age, chickens were weighed prior to being stunned by electronarcosis and euthanized by bleeding. On each animal, a 10 ml blood sample, a 10 cm segment of the ileum measured from the ileum-caecal junction, as well as the whole caecum were collected. All samples were sent on ice to the laboratory for immediate processing. The *in vivo* experiment was replicated once more with a distinct lot of birds.

### Sample Treatment

Caecal matter was collected from the caecum. A 1 g portion was used for the enumeration of *C. jejuni* while another 1 g was flash-frozen in liquid nitrogen and kept at -80°C for DNA extraction (Thibodeau et al., 2015b).

Blood samples were kept 1 h at room temperature and then centrifuged at 100 × *g* for 15 min. The supernatant was collected and divided into two distinct samples that were kept at -20°C. One sample was used to determine the seric IgY concentrations and the other one was sent to the diagnostic laboratory of the Veterinary medicine faculty for the determination of the total glutathione peroxidase activity (GPX).

The 10 cm ileal segment was opened longitudinally and emptied of its contents with a gloved finger. A sterile microscopic glass slide was then used to scrape off the mucus which was resuspended in 10 ml of cold PBS. The mucus suspension was then kept at -20°C until used for the determination of the intestinal mucus IgA concentration.

### Seric IgY and Intestinal IgA Levels

The concentration of the total seric IgY and intestinal IgA was assessed by ELISA using commercial kits from Bethyl laboratories (Bethyl Laboratories, Montgomery, AL, USA) for eight chickens per experimental group. Protocols were performed according to the manufacturer recommendations. Serum samples were used at a dilution of 1:50,000 while the intestinal samples were used at a dilution of 1:20. For IgY, the secondary antibody was used at a dilution of 1:20,000 while a dilution of 1:40,000 was used for the IgA.

### DNA Extraction, Amplicon MiSeq Sequencing, and Bioinformatics

Total DNA was extracted from all the caecal samples kept at -80°C using a combination of a beads-beating lysis and phenol–chloroform purification as previously described (Thibodeau et al., 2015b). A sample without caecal matter was extracted at the same time as a negative control for use in the downstream molecular biology analysis. DNA concentration was assessed using the Qubit BR assay (Fisher Scientific, Ottawa, ON, Canada). The DNA samples were diluted to a concentration of 10 ng/μl, separated in aliquots, and kept at -20°C until use.

A survey of the chicken caecal microbiota was performed by amplifying and sequencing the V4 region of the 16S rRNA gene from the DNA extracted from the chicken caecal samples, according to Illumina’s “16S Metagenomic Sequencing Library Preparation” guide (Part # 1504423 Rev. B). In each experimental group, the DNA extracted from the ceacum of eight chickens was used. The 16S rRNA gene PCR mastermix (25 μl final volume per reaction) consisted of 1x KAPA HiFi HotStart ReadyMix (Kappa Biosystems, Willington, MA, USA), 600 nM of each primer (Caporaso et al., 2012), 0.4 mg/ml BSA, and 12.5 ng of DNA. The following PCR cycling conditions were used: initial denaturation at 95°C for 5 min followed by 25 cycles consisting of a 30 s denaturation at 95°C, a 30 s annealing at 55°C, and an elongation of 180 s at 72°C that ended with a final elongation of 10 min at 72°C. The amplicons were purified using AMPure XP beads (Beckman Coulter, Brea, CA, USA) according to the manufacturer protocol.

Purified amplicons were barcoded using the Nextera XT Index Kit (Illumina, San Diego, CA, USA) using an eight cycles PCR: initial denaturation at 95°C for 3 min, followed by cycles consisting of a 30 s denaturation at 95°C, a 30 s annealing at 55°C, and an elongation of 30 s at 72°C, and then by a final elongation of 5 min at 72°C. Reactions consisted of 1x KAPA HiFi HotStart ReadyMix (Kappa Biosystems), 2.5 μl of each index, and 5 μl of the purified 16S rRNA gene amplicons. The index PCR was also purified using AMPure XP beads (Beckman Coulter) according to the manufacturer protocol. The purified indexed amplicons were quantified with Qubit HS kit (Fisher Scientific) and diluted to 2 ng/μl. Five microliters of each index PCR was pooled in a single tube and sent to NRC Montréal for MiSeq Sequencing, using the Miseq reagent 500 V2 kit (Illumina) for a 2x 250 bp length, as specified by Illumina.

Raw demultiplexed reads were received from the sequencing center and processed using Mothur version 1.38 (Schloss et al., 2009), following the online MiSeq SOP^1^. Prior to OTU clustering, the negative control sample and some samples containing too few or suspicious reads were removed.

To determine OTUs, reads were clustered using Vsearch with the AGC method at the 0.03 level. From this point on, results were analyzed separately for birds raised during the first and second replicate experiment. Reads were aligned and classified using the Silva database (version 123).

For diversity analysis, reads were subsampled or rarefied to the lowest number of reads found in a single sample. The following alpha-diversity indices were computed and compared across conditions: coverage, Sobs, Inverse Simpson, Shannon, and Shannon’s evenness. Beta-diversity analysis was performed by comparing the bird’s microbiota structure using Yue and Clayton diversity index and analyzed by AMOVA and HOMOVA. The composition was analyzed by LDA effect size (LEFSE) (Segata et al., 2011) according to the experimental conditions. The bird’s microbiota compositions were also compared by LEFSE using the phylotype approach. For LEFSE, only significant OTU with a LDA score over 2 were reported. Raw reads for each chicken caecal microbiota analyzed in this study are available through the NCBI SRA database under accession SRP094491.

### *C. jejuni* Enumeration and PCR

For all chickens, a 1 g of fresh caecal matter was homogenized in 9 ml of a tryptone-salt solution composed of 0.1% (w/v) tryptone (LabM, Heywood, UK) and 0.85% (w/v) NaCl (Fisher Scientific). For the birds that were not inoculated with *C. jejuni*, 100 μl of this suspension was plated on mCCDA (LabM) and immediately incubated at 42°C in a microaerobic atmosphere using chemical gas pack generators (Oxoïd, Ottawa, ON, Canada) (Macé et al., 2015). This protocol was also used for confirming the absence of *C. jejuni* in all birds at day 12.

For the *C. jejuni* inoculated birds, the caecal suspensions were diluted up to 10^6^ and the last four dilutions were plated on mCCDA (LabM) and immediately incubated at 42°C in a microaerobic atmosphere (Oxoïd). The positive control used for monitoring the adequate *C. jejuni* growth was *C. jejuni* strain ATCC 33291. After 48 h of incubation, typical colonies were enumerated and the results were log 10 transformed to assess the effect of selenium-yeast supplementation on the chicken *C. jejuni* carriage.

To corroborate the colonization status determined by culture, all culture negative and culture positive *C. jejuni* samples were confirmed by PCR (Yamazaki-Matsune et al., 2007). The PCR mix (25 μl) was composed of primers (C412F at 200 nM, C1228R at 200 nM, C-1 at 800 nM, and C-3 at 800 nM), 1 unit of Taq DNA polymerase (Bio Basic, Markham, ON, Canada), MgSO_4_ at 2 mM, and dNTPs at 200 mM. PCR amplicons were visualized on a 1% agarose (Fisher Scientific) gel stained with Sybrsafe (Fisher Scientific). Positive control consisted of DNA extracted from *C. jejuni* strain ATCC 33291 while the negative control contained no DNA.

### Statistical Analysis

Comparison of the chicken’s body weight, *C. jejuni* colonization levels, seric total GPX activity, seric IgY concentrations, intestinal IgA concentrations, and alpha-diversity indices were analyzed in GraphPad Prism 5 (GraphPad Software, La Jolla, CA, USA). Prior to selecting the correct statistical analysis, the distribution of the data was inspected. When the normality was confirmed, data were analyzed using parametric tests. Otherwise, non-parametric analyses were conducted. An alpha of 0.05 was set to assess significance. Results, with the exception of the microbiota, were analyzed separately for the first or second replicate according to the selenium-yeast or *C. jejuni* status before being pooled for the analysis of the global effect observed in this study.

## Results

### Chicken Body Weight

After 35 days of growth, no significant difference (*p* > 0.05) was observed in regards to the chicken final body weight, though the use of selenium-yeast consistently yielded chickens with higher mean body weight values in both replicates (**Table 1**).

### Seric GPX Levels

Seric GPX levels were observed to be significantly lower (*p* < 0.05) for the chickens fed selenium-yeast (**Table 2**). No significant difference (*p* > 0.05) was observed for chickens inoculated with *C. jejuni*, although consistent lower mean values for the inoculated chickens were observed. A large inter-individual variation was also observed, as exemplified by the high standard deviation values.

### Immunoglobuline Concentrations

No significant difference (*p* > 0.05) was measured regarding total seric IgY concentrations (**Table 3**). For the intestinal IgA recovered from the ileal mucus layer (**Table 4**), significantly lower concentrations were observed in selenium-yeast supplemented chickens, but only for the second experimental replicate (*p* < 0.05). Similarly, IgA concentrations were significantly lower in chickens not inoculated with *C. jejuni* (*p* < 0.05), but only for the second experimental replicate.

### Microbiota

A total of 8,438,914 sequences were obtained after assembly. Prior to OTU clustering, a total of 4,978,059 sequences remained, representing 308,896 unique sequences. The two negative controls included in this study contained 260 and 427 sequences. Two chicken caecal samples returned numbers of sequences similar to the negative controls (328 and 348) and were discarded from the analysis. Two more samples, originating from chickens not inoculated with *C. jejuni*, returned unexpectedly high numbers of reads classified as *Campylobacter*, in similar proportions to all other samples originating from inoculated birds. Further PCR analyses confirmed the samples negative for *C. jejuni* while all *C. jejuni* inoculated birds returned a strong PCR signal (data not shown); these samples were removed from the analysis. After this, the lowest number of sequences in a chicken sample was 14,482 while the highest was 161,901.

Alpha-diversity indices (**Table 5**) were first compared based on the use of selenium-yeast in the feed or the inoculation with *C. jejuni*. Coverage over 97% was observed in all treatments. In experimental replicate 2, the use of yeast-selenium significantly increased (*P* < 0.05) the coverage and richness (Sobs) of the bird’s microbiota but this was not observed in experimental replicate 1. Richness (Sobs) was significantly different between the two experimental replicates regardless of the experimental condition (*p* < 0.001), with mean values of 435 (±130) and 687 (±225) for experimental replicate 1 and 2, respectively. The observed mean Shannon indices (3.50 ± 0.15 and 3.62 ± 0.25 for experimental replicates 1 and 2, respectively) and Shannon Evenness indices (0.58 ± 0.03 and 0.56 ± 0.03 for experimental replicates 1 and 2, respectively) were also significantly different (*p* = 0.001) between replicates.

**FIGURE 1:**
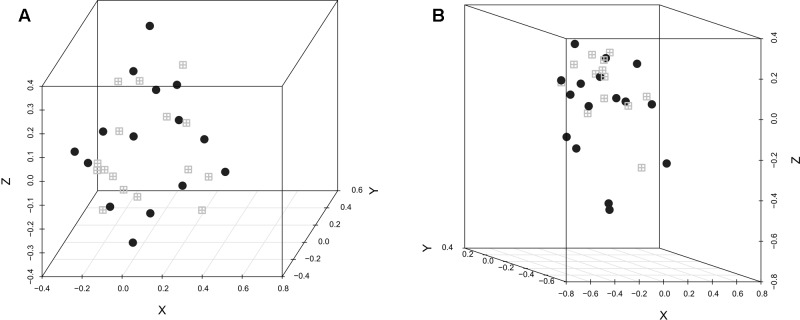
**NMDS graphic representation of caecal microbiota diversity according to the use of selenium-yeast or not.** The gray windows represent the chickens that did not receive selenium-yeast while the black circles represent the chickens that did. **(A)** Replicate 1: AMOVA: *p* > 0.05; HOMOVA: *p* > 0.05; lowest stress: 0.091; *R*^2^: 0.939. **(B)** Replicate 2, AMOVA: *p* > 0.05; HOMOVA: *p* > 0.05; lowest stress: 0.098; *R*^2^: 0.958.

**FIGURE 2:**
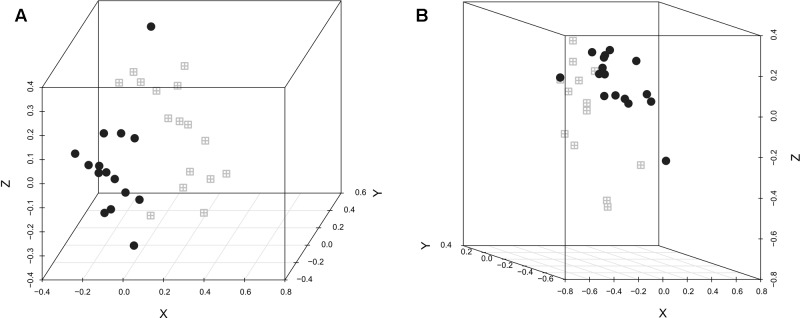
**NMDS graphic representation of caecal microbiota diversity according to *C. jejuni* inoculation or not.** The gray windows represent the chickens that were not inoculated with *C. jejuni* while the black circles represent the chickens that were. **(A)** Replicate 1, AMOVA: p < 0.001; HOMOVA: p > 0.05; lowest stress: 0.091; R2: 0.939. **(B)** Replicate 2, AMOVA: p < 0.001; HOMOVA: p > 0.05; lowest stress: 0.098; R2: 0.958.

The difference in the microbiota structure according to the experimental conditions was investigated. In both replicates, selenium-yeast supplementation (**Figure 1**) did not influence the microbiota structure, while the inoculation with *C. jejuni* (**Figure 2**) did influence the caecum bacterial community. When analyzing all chickens together, the bacterial community structure was significantly different between the experimental replicates (AMOVA *p* < 0.001, HOMOVA *p* = 0.2).

The LEFSE analysis identified OTUs that were consistently associated with the inoculation of *C. jejuni* or the use of selenium-yeast. When using *C. jejuni* inoculation as a class and selenium-yeast as a subclass, 75 OTUs were identified in replicate 1 and 71 OTUs in replicate 2. Of these, only 17 OTUs were consistently associated in both replicates (**Table 6**). On the contrary, when using selenium-yeast as the class and the inoculation with *C. jejuni* as a subclass, for replicate 1, five OTUs were identified compared to two OTUs in replicate 2. For selenium-yeast, no OTU were common to both replicates. All LEFSE results, broken down per replicate, are available in the Supplementary Material.

Using phylotype analysis with genera as the cutoff (mothur taxlevel = 1), 23 OTUs were found to be associated with *C. jejuni* inoculation for experimental replicates 1 and 2, and the six following OTUs were found for both replicates: unclassified_ *Ruminococcaceae*, *Eisenbergiella*, *Tyzzerella_3*, and *[Eubacterium]_hallii_group* were associated with the non-inoculated birds while *Lachnoclostridium* and *Campylobacter* were associated with the inoculated birds. For supplementation with selenium-yeast, two OTUs were identified in each of the experimental replicates, but none were shared. Apart from the obvious association of sequences from the *C. jejuni* lineage (*Proteobacteria*; *Epsilonproteobacteria; Campylobacterales*; *Campylobacteraceae*) with *C. jejuni* inoculated chickens, no other association with treatments were found when using different taxonomical levels (mothur taxlevel 2, 3, 4 or 5) in LEFSE analysis. All LEFSE phylotype results, broken down per replicate, are available in the Supplementary Material.

### *C. jejuni* Colonization

In experimental replicate 1, the *C. jejuni* caecal concentrations were found to be slightly but significantly higher for the chicken supplemented with selenium-yeast (**Table 7**) (*p* < 0.05). The opposite effect was observed for experimental replicate 2. When all the birds from the two replicates were used together in the same analysis, no significant difference remained (*p* > 0.05). Confirmation of the presence of *C. jejuni* in the inoculated chickens was carried out by PCR and culture, which confirmed that all inoculated chickens were infected by *C. jejuni* while the all the non-inoculated chickens were not.

## Discussion

The aim of this study was to assess the impact of a selenium-yeast feed additive on some chicken health related parameters as well as on the caecal colonization of *C. jejuni*. Based on the observations made during this study, it cannot be concluded that the use of yeast-selenium positively modulated the parameters measured in healthy chickens.

Nevertheless, a non-significant trend of increased performances in terms of weight gain was observed in the present study, an observation also reported by some other studies (Jensen and Mc, 1960; Choct et al., 2004; Markovic et al., 2014; Suchy et al., 2014). The present study was conducted in a level 2 facility that allows precise control of the chickens’ rearing environment, therefore reducing confounding factors and allowing the study of individual conditions. Raising chickens with high biosecurity clearly maximizes chicken growth, limiting the chances that a supplement will further increase the performance of chickens.

In this study, it was also observed that the use of selenium-yeast could lower slightly, but significantly seric GPX levels in both biological replicates as well as the intestinal IgAs in replicate 2 and when combining both replicate results. We also observed a high and unexpected variation between individual samples. This observation is in opposition with the current literature where the use of selenium is usually associated with increased levels of immunoglobulins and GPX, factors associated with healthy chickens (Chen et al., 2014; Placha et al., 2014; Boostani et al., 2015). Under our experimental settings, the exact mechanisms driving these unexpected results remained unidentified.

This study was also the first to look at the potential impact of selenium-yeast on the chicken microbiota. Despite the observation of some biological modifications due to selenium-yeast supplementation, no impact on both alpha and beta diversity was observed. This study was conducted under controlled conditions where the available bacteria that might contribute to the development of the chicken microbiota are expected to be limited and quite different from the ones field chickens could encounter. Our experiment should therefore be replicated in commercial settings to fully understand the effects of selenium-yeast supplementation of the chicken microbiota.

When using plate counts, no significant differences in *C. jejuni* colonization were observed when the selenium-yeast was used as a feed-additive, which is in agreement with the lack of modulation of the caecal microbiota observed in selenium-yeast supplemented chickens. Under experimental conditions less favorable to chicken health, selenium supplementation was reported to be beneficial for chicken (Xu et al., 2015). It has been observed that the competition for zinc within the chicken microbiota plays a role in the effective colonization of *C. jejuni* (Gielda and DiRita, 2012). It could therefore be interesting to monitor *C. jejuni* colonization under various levels of competition for selenium acquisition. Using different *C. jejuni* strains might also lead to different results since the impact of selenium on the overall *C. jejuni* population has yet to be fully characterized.

*Campylobacter jejuni* colonization of the caecal microbiota was confirmed here to slightly reorganize the caecal microbiota, in keeping with past results obtained using the same *C. jejuni* strains and with birds raised within similar parameters (Thibodeau et al., 2015b). However, when analyzing the shift in microbiota composition associated with *C. jejuni* colonization, different results were obtained. In our previous study, *C. jejuni* colonization was associated with changes in the relative abundance of the genera *Streptococcus*, *Blautia*, *Anaerofilum*, *Faecalibacterium*, *Clostridium*, *Coprobacillus*, and *Anaeroplasma*, which was not the case in the current study.

The few studies that evaluated the impact of *C. jejuni* colonization on the chicken caecal microbiota all showed that the microbiota structure is somewhat affected, but different conclusions were reached when comparing microbiota compositional changes (Johansen et al., 2006; Sofka et al., 2015; Saint-Cyr et al., 2016). This might be due to the use of different DNA extraction methods, different 16S rRNA gene regions being sequenced, and different bioinformatics pipelines used to process raw sequences (de la Cuesta-Zuluaga and Escobar, 2016). Even when taking in account technical discrepancies and the differences observed between replicates in the present study, these results, when taken together, clearly indicate that the existing chicken caecal microbiota reacts somewhat to the presence of *C. jejuni*, regardless of the initial microbiota of the chicken. This indicates a potential commensal lifestyle for *C. jejuni* that would act as a super colonizer. This strongly suggests that the on-farm control of *C. jejuni* via modification of the caecal microbiota only is a titan’s task, which is reflected in the lack of recent significant advances in the control of the colonization of chicken by *C. jejuni* using feed additives.

## Conclusion

We observed here that selenium-yeast supplementation modified, but did not improve the general health of broiler chickens at slaughter age and that selenium-yeast supplementation could even be somewhat detrimental when chickens are raised in controlled conditions maximizing their health. These changes were not associated with any modification of the caecal microbiota. This suggests that the microbiota is not always linked to the animal’s health parameters and that healthy animal do not exhibit a common and defined microbiota, highlighting the need for further studies to define a truly healthy microbiota. Moreover, the use of selenium-yeast supplementation did not modify the chicken colonization by *C. jejuni.* This study also confirmed that *C. jejuni* colonization can slightly modify the caecal microbiota, an observation in line with the potential commensal lifestyle of *C. jejuni*. This study also illustrated the importance of true biological replicates when studying the chicken intestinal microbiota, especially when the observed changes are subtle.

## Author Contributions

AT designed the experiments, did all experimentations, analyzed all results, discussed the results and wrote the manuscript. AL, EY, and PF designed the experiments, discussed the results and revised the manuscript. GL-G did some experimentations, discussed the results and revised the manuscript.

## Conflict of Interest Statement

The authors declare that the research was conducted in the absence of any commercial or financial relationships that could be construed as a potential conflict of interest.

## Footnotes

1 https://www.mothur.org/wiki/MiSeq_SOP
